# Data for the synthesis, characterization, and use of xerogels as adsorbents for the removal of fluoride and bromide in aqueous phase

**DOI:** 10.1016/j.dib.2022.108138

**Published:** 2022-04-08

**Authors:** Nahum Andres Medellin-Castillo, Elizabeth Diane Isaacs-Páez, Liliana Giraldo-Gutierrez, Juan Carlos Moreno-Piraján, Itzia Rodríguez-Méndez, Simón Yobanny Reyes-López, Jaime Reyes-Hernández, Sonia Judith Segovia-Sandoval

**Affiliations:** a Centro de Investigación y Estudios de Posgrado, Facultad de Ingeniería, Universidad Autónoma de San Luis Potosí, San Luis Potosí 78290, Mexico; b División de Ciencias Ambientales, Instituto Potosino de Investigación Científica y Tecnológica, A.C., San Luis Potosí 78216, Mexico; c Departamento de Química. Facultad de Ciencias, Universidad Nacional de Colombia, Sede Bogotá. Carrera 30 No. 45-03, Bogotá, Colombia; d Departamento de Química, Facultad de Ciencias, Universidad de los Andes, Carrera 1 este No 18A-10, Bogotá, Colombia; e Instituto de Ciencias Biomedicas, Universidad Autonoma de Ciudad Juarez, Cd. Juarez, Chihuahua 32300, México; f Facultad de Enfermería y Nutrición, Universidad Autónoma de San Luis Potosí, San Luis Potosí 78240, México

**Keywords:** Xerogels, Melamine, Colloidal polymerization, Fluoride and bromide ions, Adsorption

## Abstract

Groundwater with high fluoride concentrations has been recognized as one of the serious concerns worldwide. Besides, the fluoride released into the groundwater by slow dissolution of fluoride-containing rocks, various industries also contribute to fluoride pollution [1]. Excess intake of fluoride leads to various health problems such as dental and skeletal fluorosis, cancer, infertility, brain damage, thyroid diseases, etc. [2]. On the other hand, bromide is naturally present in surface and groundwater sources. However, during the chlorination process, bromide can be oxidized to HOBr, which can react with natural organic matter in water to form brominated organic disinfection byproducts, which are very harmful to human health [3]. Among various methods for water treatment, the adsorption process has been widely used and seems to be an efficient and attractive method for the removal of many contaminants in water, such as anions, in terms of cost, simplicity of design, and operation [4], [5]. In the past years, xerogels and carbon xerogels, a new type of adsorbents, which are synthesized by the sol-gel polycondensation of resorcinol and formaldehyde, have gained attention due to their moldable texture and chemical properties [6]. Moreover, melamine addition in resorcinol and formaldehyde xerogels adds basic groups on its surface, favouring Lewis acid-base interactions between xerogels and other components by adsorption [7].

In this data article, the synthesis of three resorcinol-formaldehyde (R/F) xerogels with an increasing amount of melamine (M) was carried out by colloidal polymerization (molar ratios of M/R = 0.5, M/R = 1.0, and M/R = 2.0). Additionally, samples of M/R = 0.5 xerogel were carbonized at 400, 450, and 550 °C under an inert atmosphere to increase their specific area. Organic and carbon xerogels obtained were characterized by FTIR, TGA, SEM, Physisorption of N_2_, and the pH at the point of zero charge (pH_PZC_). All organic xerogels were also tested as adsorbents on the removal of fluoride and bromide ions from aqueous phase. The Freundlich, Langmuir, and Radke-Prausnitz isotherm models were applied to interpret the experimental data from adsorption equilibrium. Additionally, the data of the mass of the xerogel needed to remove fluoride and bromide from groundwater and fulfill the maximum concentration levels are also included.

## Specifications Table

**Table:** 

Subject	Environmental science, Material science, Chemistry
Specific subject area	Environmental engineering, Wastewater remediation
Type of data	Table, Image, Text, and Figure
How data were acquired	Xerogels were synthesized using a colloidal polymerization method with different additions of melamine. Xerogels were characterized by Fourier transform infrared spectrophotometer (Thermo Scientific, Nicolet iS10) wavelength 4000 to 500 cm−1 and a 16 cm^−1^ resolution; Scanning Electronic Microscopy (FEI Quanta 200); Thermogravimetric analysis (Setaram Instruments, Setsys Evolution TG/DSC) were performed under N_2_ atmosphere at a heating rate of 10 °C/min; The textural properties such as specific area, pore volume, and average pore diameter were determined using adsorption/desorption isotherms of N_2_ at 77 K (Micromeritics, ASAP 2020); specific area was obtained by using Brunauer-Emmett-Teller method (BET), and pH_PZC_. The adsorption equilibrium experiments were carried out in batch mode at pH=5 and room temperature (25 ± 1 °C). The pH was monitored with a digital pH meter and adjusted by adding drops of 0.01 or 0.1 N NaOH or HNO_3_ solutions.
	The experimental adsorption equilibrium data for fluoride and bromide were analyzed using the adsorption isotherm models of Freundlich, Langmuir, and Radke-Prausnitz.
Data format	Raw Analyzed
Parameters for data collection	Xerogels were synthesized using resorcinol (R)-formaldehyde (F) and melamine (M) by colloidal polymerization with the following molar ratios of M/R = 0.5, M/R = 1.0, and M/R = 2.0. Also, samples of M/R = 0.5 xerogel were carbonized at 400, 450, and 550 °C under an inert atmosphere. Organic and carbon xerogels obtained were characterized by FTIR, TGA, SEM, Physisorption of N_2_, and the pH at the point of zero charge (pH_PZC_). All xerogels were also tested as adsorbents on the removal of fluoride and bromide ions from aqueous phase. The mass of the xerogel needed to remove fluoride and bromide from groundwater and fulfill the maximum concentration levels was estimated.
Description of data collection	Xerogels were synthesized using the methodology proposed by Muehlemann et al. [9]. The xerogels M/R = 0.5 were carbonized in a horizontal tubular furnace Carbolite model 12/65/550 under a N_2_ flow at 400, 450, and 550 °C for 2 h. The equilibrium adsorption experiments were carried out in a batch adsorber using 0.02 g of carbonized xerogel, 50 mL of fluoride, or bromide solution with different initial concentrations ranging from 10 to 100 mg L^−1^. The experiments were carried out at constant pH. The mass of fluoride or bromide adsorbed per gram of adsorbent (q), was obtained using a mass balance equation. The adsorption equilibrium data were fitted by Langmuir, Freundlich and Prausnitz-Radke isotherm models.The bromide and fluoride concentration in aqueous solution was measured by a potentiometric method with a bromide or fluoride ion-selective electrode.
Data source location	Centro de Investigación y Estudios de Posgrado, Facultad de Ingeniería, Universidad Autónoma de San Luis Potosí, Av. Dr. M. Nava No. 8, Zona Universitaria, San Luis Potosí, SLP, 78,290, Mexico.
Data accessibility	The data sets are deposited in Mendeley Data https://data.mendeley.com/datasets/gvjd33sw57/2

## Value of the Data

• The data provided is valuable to develop xerogels of Resorcinol/Formaldehyde/Melamine by a simple method of colloidal polymerization.
• Melamine addition increases the basic groups content, which improves fluoride and bromide ions adsorption onto xerogels.
• These data show that fluoride and bromide adsorption on xerogels was not mainly dependent on the textural properties of adsorbents.

## 1 Data Description

**Fig. 1:**
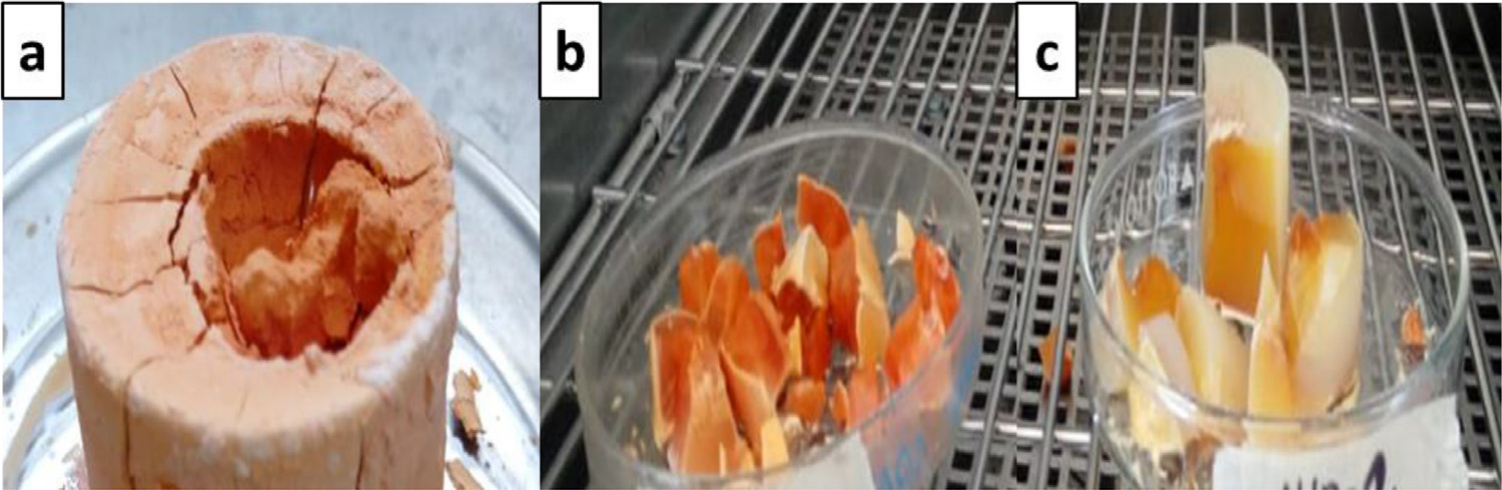
Physical appearance of organic xerogels a) M/R = 0.5, (b) M/R = 1.0, (c) M/R = 2.0.

**Fig. 2:**
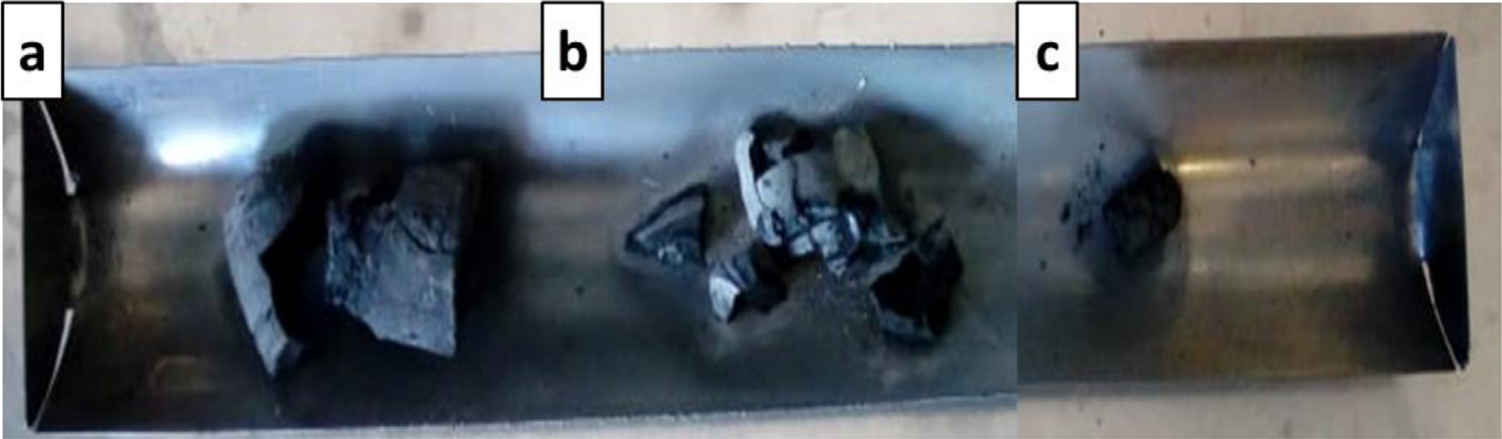
Physical appearance of M/R = 0.5 xerogels carbonized at (a) 400 °C, (b) 450 °C, and (c) 550 °C.

**Fig. 3:**
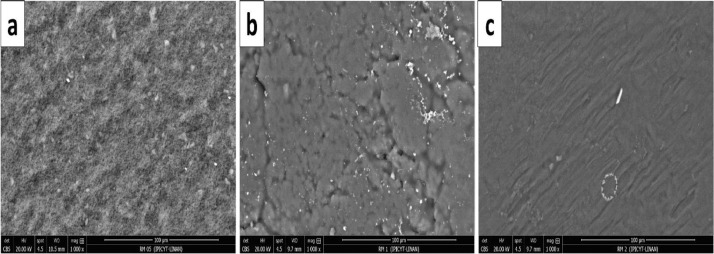
SEM images of organic xerogels a) M/R = 0.5, (b) M/R = 1.0 and, (c) M/R = 2.0.

**Fig. 4:**
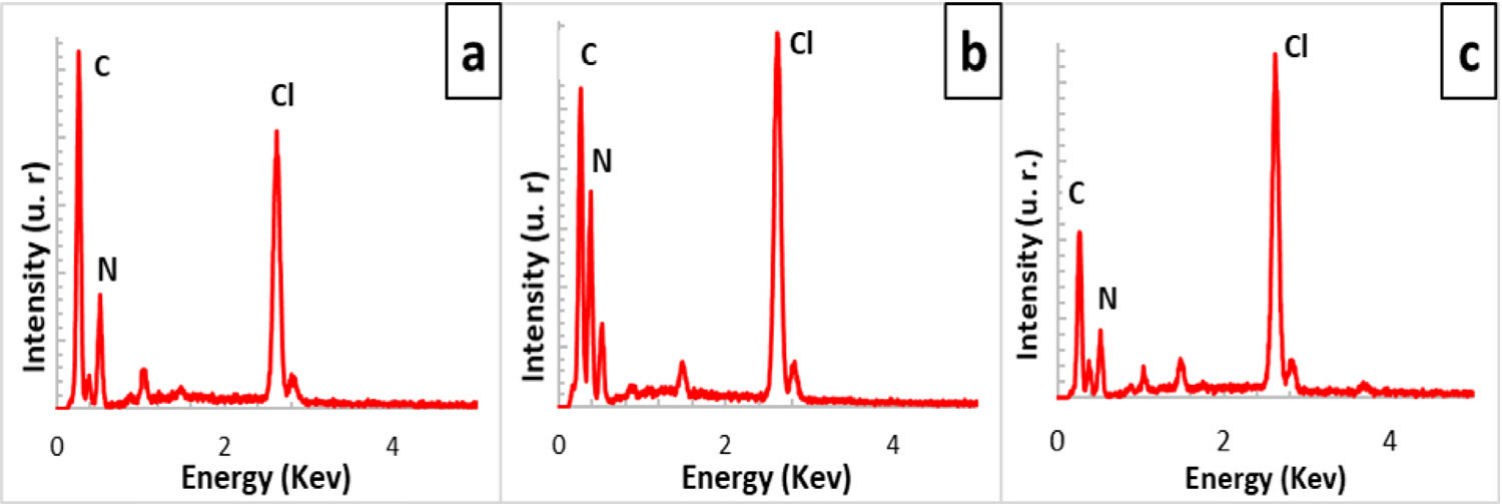
EDS microanalysis of xerogels; a) M/R = 0.5, (b) M/R = 1.0 and, (c) M/R = 2.0.

**Fig. 5:**
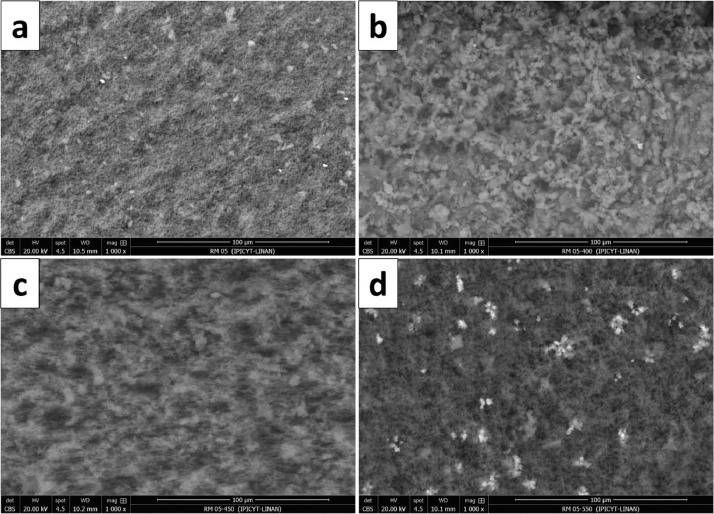
SEM images of M/*R* = 0.5 xerogel (a) organic xerogel, (b) carbonized at 400 °C, (c) carbonized at 450 °C, and (d) carbonized at 550 °C.

**Fig. 6:**
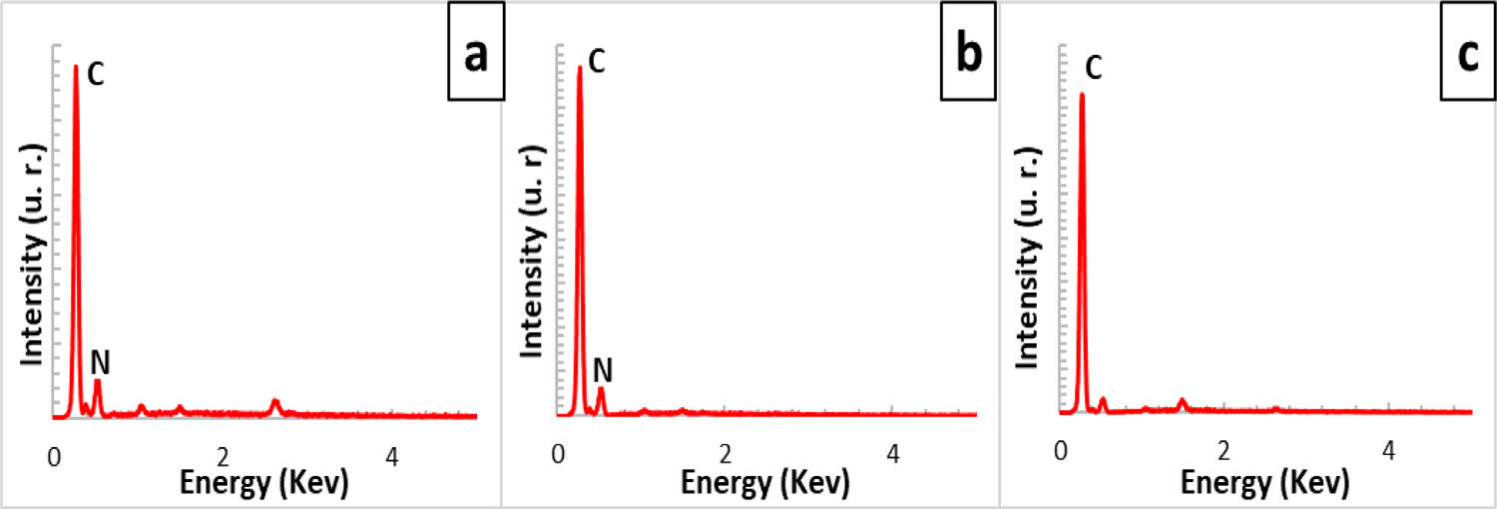
EDS microanalysis of carbon xerogels; (a) M/R = 0.5 400, (b) M/R = 0.5 450, and (c) M/R = 0.5 550.

Fig. 1 shows the physical appearance of synthesized organic xerogels at different melamine-resorcinol molar relations (M/R = 0.5, M/R = 1.0, and M/R = 2.0) after 48 h drying. Fig. 2 shows the physical appearance of M/R = 0.5 xerogels carbonized at 400, 450, and 550 °C. Fig. 3, Fig. 4 exhibit the surface morphology (SEM images) and the elemental chemical composition determined by Energy Disperse Spectrum (EDS) microanalysis of M/R = 0.5, M/R = 1.0, and M/R = 2.0 xerogels, respectively. Fig. 5, Fig. 6 show the surface morphology (SEM images) and the EDS microanalysis of M/*R* = 0.5 xerogel carbonized at three different temperatures: 400, 450, and 550 °C, respectively. Carbon xerogels samples were label as M/R = 0.5 400, M/R = 0.5 450, and M/R = 0.5 550. Table 1 exhibits the textural properties (specific area, total pore volume, and mean pore diameter) and pH_PZC_ of xerogels and carbon xerogels samples.

**Table 1:** Textural properties and pH_PZC_ of organic and carbon xerogels.

Xerogel sample	S_BET_^a^(m^2^/g)	V_P_^b^(cm^3^/g)	d_P_^c^(nm)	pH_PZC_
M/R 0.5	16	0.062	15.6	3.0
M/R 1.0	6	0.026	14.6	3.1
M/R 2.0	2	0.014	20.7	3.2
M/R 0.5 400	12	0.071	25	4.0
M/R 0.5 450	237	0.142	7.7	6.0
M/R 0.5 550	378	0.134	7.7	5.9

a Specific area.

b Total pore volume.

c Mean pore diameter.

**Fig. 7:**
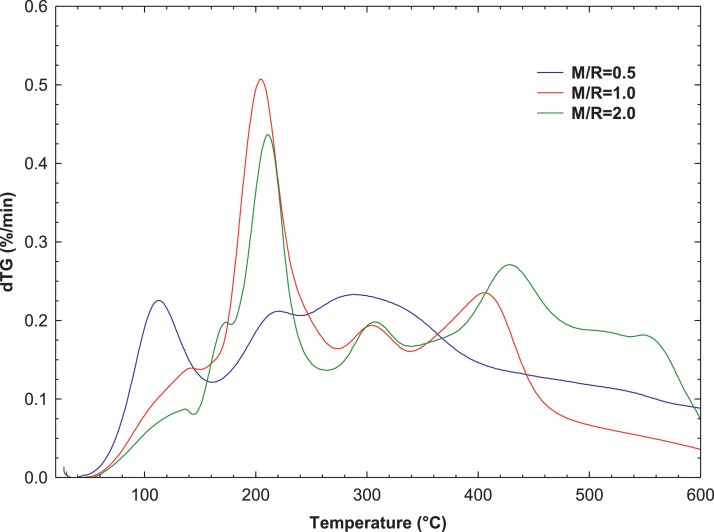
Thermogravimetric curves for M/R = 0.5, M/R = 1.0, and M/R = 2.0 organic xerogels under N_2_ atmosphere.

**Fig. 8:**
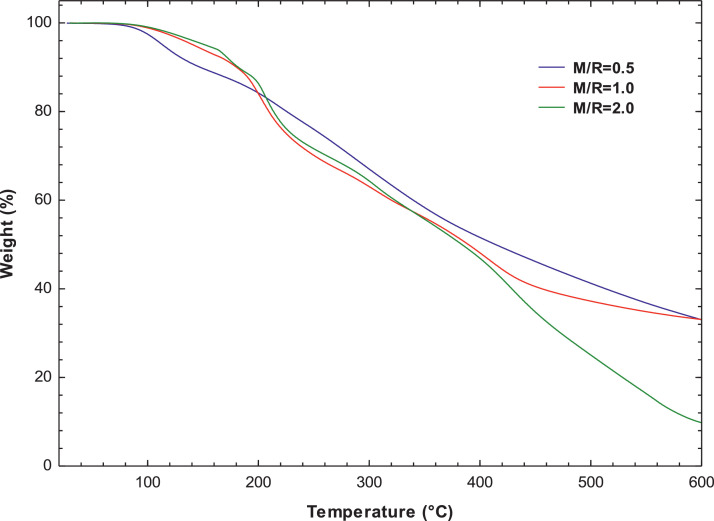
Thermogravimetric curves of mass loss percentages for M/R = 0.5, M/R = 1.0, and M/R = 2.0 xerogels.

**Fig. 9:**
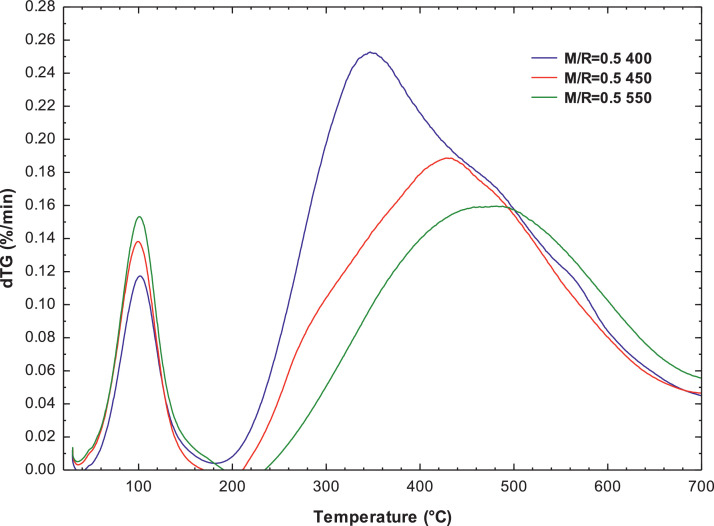
Thermogravimetric curves for M/R = 0.5 400, M/R = 0.5 450, M/R = 0.5 550 carbon xerogels under N_2_ atmosphere.

**Fig. 10:**
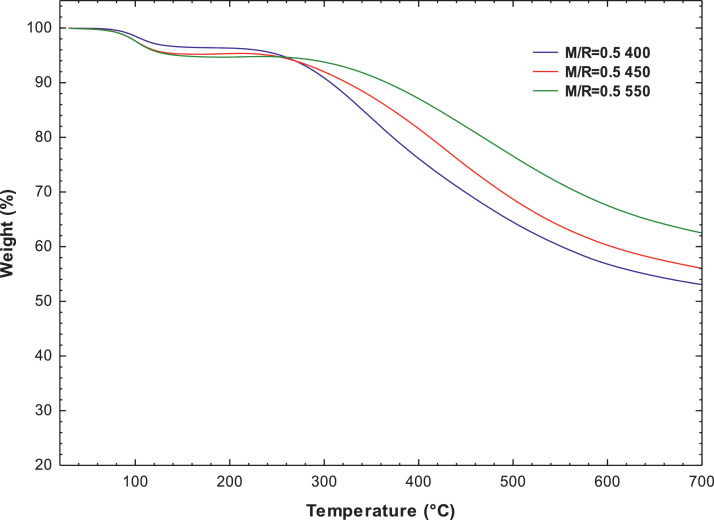
Thermogravimetric curves of mass loss percentages for the samples of carbon xerogels M/R = 0.5 400, M/*R* = 0.5 450, and M/*R* = 0.5 550.

**Fig. 11:**
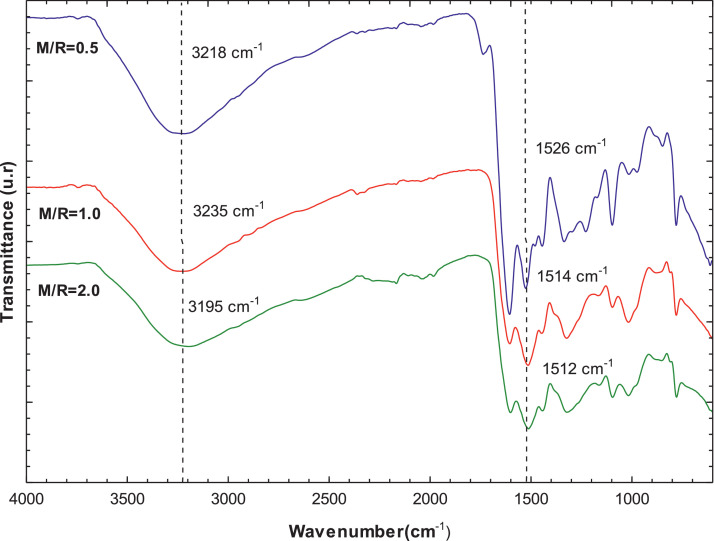
FTIR spectra of organic xerogels at different melamine-resorcinol molar relation (M/R = 0.5, M/*R* = 1.0, and M/*R* = 2.0).

Fig. 7, Fig. 8 present the thermogravimetric profile of speed mass loss and mass loss percentage obtained for M/R = 0.5, M/R = 1.0, and M/R = 2.0, respectively. Fig. 9, Fig. 10 show the thermogravimetric profile of speed mass loss and mass loss percentage obtained for M/R = 0.5 400, M/R = 0.5 450, and M/R = 0.5 550, respectively. Fig. 11 exhibit the FTIR spectrums of M/R = 0.5, M/R = 1.0, and M/R = 2.0 xerogels.

**Fig. 12:**
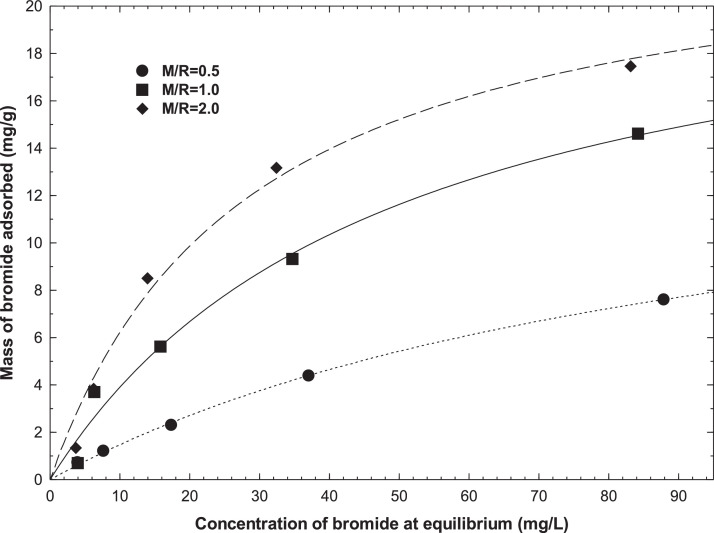
Adsorption isotherm of bromide from aqueous solution on organic xerogels at pH = 5.0 and *T* = 25 °C. The lines represent the Langmuir isotherms.

**Fig. 13:**
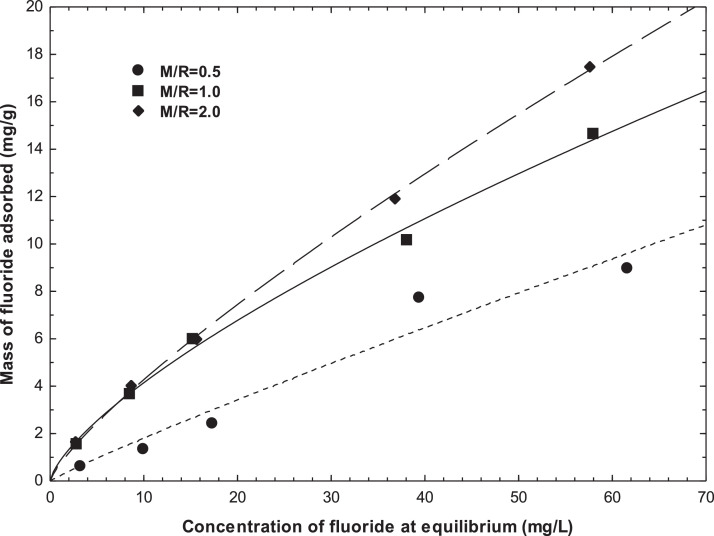
Adsorption isotherm of fluoride from aqueous solution on organic xerogels at pH = 5.0 and *T* = 25 °C. The lines represent the Freundlich isotherms.

**Fig. 14:**
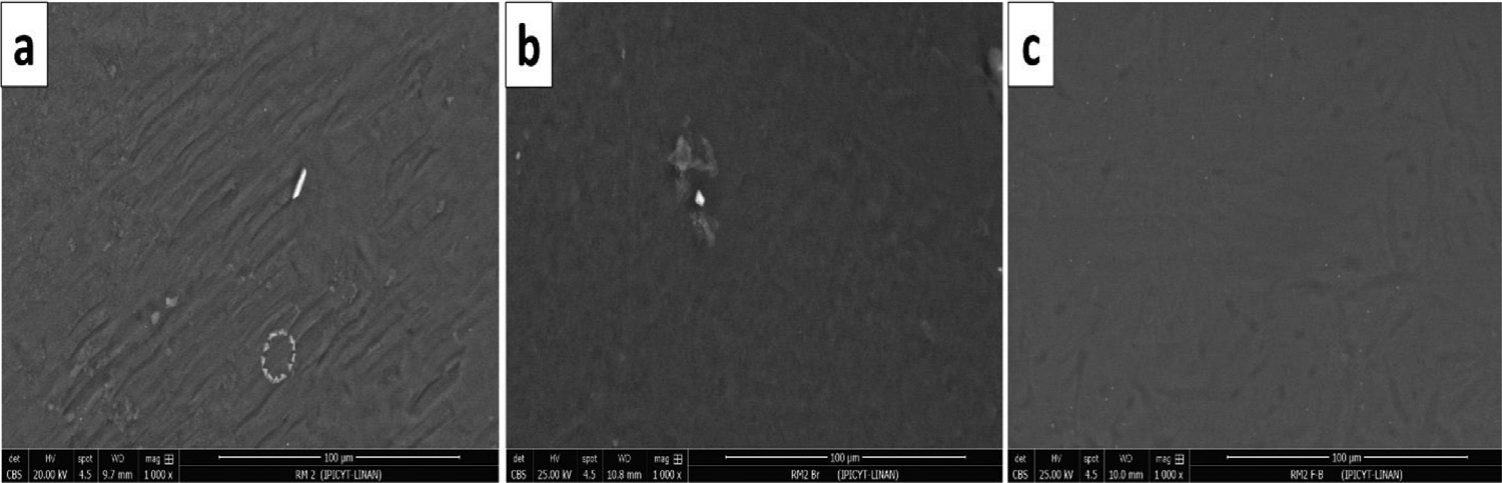
SEM images of M/R = 2.0 xerogel (a) fresh, (b) with bromide adsorbed, and (c) with fluoride adsorbed.

The isotherm model constants values and%D for bromide and fluoride adsorption on M/R = 0.5, M/R = 1.0, and M/R = 2.0 xerogels are given in Tables 2 and 3, respectively. Fig. 12 shows the adsorption isotherm of bromide from an aqueous solution on xerogels at pH=5.0 and T = 25 °C. The lines represent the Langmuir isotherms. Fig. 13 presents the adsorption isotherm of fluoride from an aqueous solution on xerogels at pH=5.0 and T = 25 °C. The lines represent the Freundlich isotherms. Fig. 14 shows the SEM images of M/R = 2.0 xerogel fresh and bromide and fluoride adsorbed.

**Table 2:** Freundlich, Langmuir, and Radke-Prausnitz isotherms parameters for adsorption of bromide on xerogels at pH = 5 and 25 °C.

No. Exp.	Freundlich a			Langmuir b			Prausnitz-Radke c			
M/R	k(mg^1–1/n^L^1/n^/g)	n	%D	q(mg/g)	k(L/mg)	%D	A(L/g)	B(L/mg)	β	%D
0.5	0.31	1.39	4.95	16.23	0.015	2.29	0.18	0.031	0.78	5.29
1.0	1.01	1.64	7.2	22.97	0.02	7.91	0.52	0.042	0.88	8.03
2.0	1.83	1.91	14.55	23.82	0.035	5.99	0.58	0.013	1.12	12.91

a $q=K_{f}C_{e}^{1/n}$ K_f_ is the Freundlich constant, n is the Freundlich constant (mg/g(L/mg)^1/n^); q is the amount of bromide or fluoride adsorbed per gram of adsorbent (mg/g), C_e_ is the equilibrium bromide or fluoride concentration (mg/L), q_m_ is the maximum adsorption capacity (mg/g), K_L_ is the Langmuir constant (L/mg), A is the constant of the Radke-Prausnitz (L/g); B is the constant of the Radke-Prausnitz (L^β^/mg^β^); β is the constant of the Radke-Prausnitz.%D is the average absolute percentage deviation (%), N is the number of experimental data points, q_iexp_ is the mass of the fluoride or bromide adsorbed at equilibrium, and q_ipred_ is the mass of the fluoride or bromide adsorbed at equilibrium predicted.

b $q=\frac{q_{m}K_{L}C_{e}}{1+K_{L}C_{e}}.$

c $q=\frac{AC_{e}}{1+{\text{BC}}_{e}^{\beta }}$^d^$\%D=(\frac{1}{N}\sum_{i=1}^{N}\left|\frac{q_{i,exp}-q_{i,pred}}{q_{i,\;exp}}\right|)\times \;100\%.$

**Table 3:** Freundlich, Langmuir, and Radke-Prausnitz isotherms parameters for adsorption of fluoride on xerogels at pH = 5 and 25 °C.

No. Exp.	Freundlich ^a^			Langmuir ^b^			Radke-Prausnitz ^c^			
M/R	K(mg^1–1/n^L^1/n^/g)	n	%D	q(mg/g)	k(L/mg)	%D	A(L/g)	B(L/mg)	β	%D
0.5	0.22	1.09	17.33	55.46	0.007	18.0	0.18	0.002	1.16	17.54
1.0	0.81	1.41	3.66	30.54	0.015	6.01	0.45	0.024	0.87	9.83
2.0	0.68	1.25	2.6	23.82	0.008	4.87	0.41	0.13	0.81	10.14

Considering the adsorption capacity of bromide and fluoride onto M/R = 2.0 xerogel shown in Figs. 12 and 13, the mass of M/R = 2.0 xerogel needed to fulfill the maximum permissible limit (MPL) of bromide and fluoride in groundwater established by WHO (2002), respectively [8], it was calculated from the mass balance of Eq. (1), as shown in Table 4.

**Table 4:** Amount of M/*R* = 2.0 xerogel material needed to fulfill the MPL of bromide and fluoride in 1 L of groundwater.

	C_0_ (mg/L)	C_f_ (mg/L)	q (mg/g)	m (g)
**Bromide**	1.0	0.4	0.33	1.8
**Fluoride**	10	1.5	0.94	9.0

**Fig. 15:**
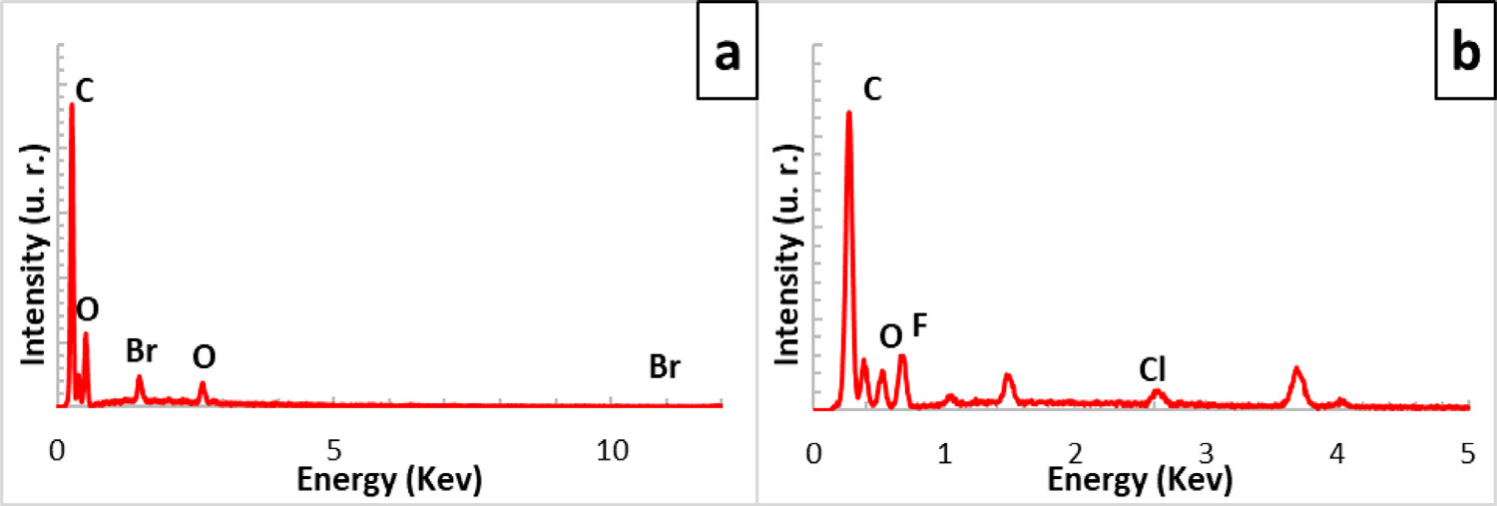
EDS microanalysis of M/R = 2.0 xerogel (a) bromide adsorbed, and (b) fluoride adsorbed.

**Fig. 16:**
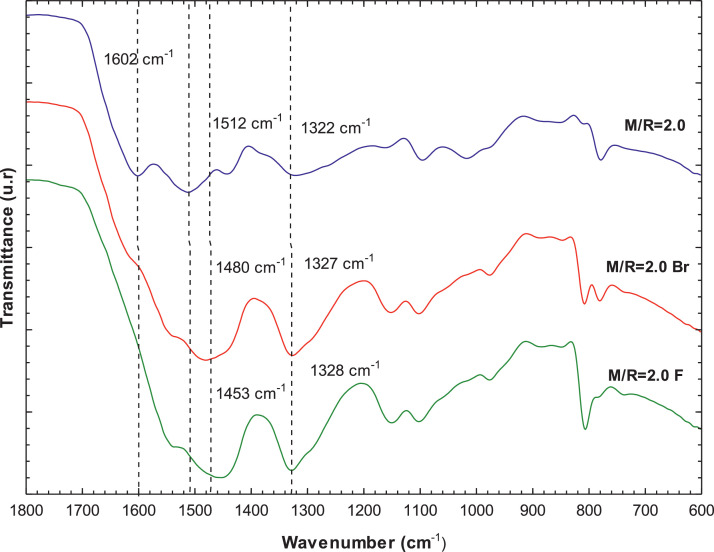
FTIR spectra of M/R = 2.0 xerogel before and after fluoride and bromide adsorption.

Fig. 14 shows the comparison of the surface morphologies (SEM images) among fresh M/R = 2.0 and M/R = 2.0 xerogels loaded with bromide and fluoride. Fig. 15 exhibit the EDS microanalysis of M/R = 2.0 xerogel with bromide and fluoride adsorbed and Fig. 16 present the FTIR spectrums of M/R = 2.0 xerogel before and after fluoride and bromide adsorption.

All raw data associated with the figures in this work can be found in the supplementary material.

All related primary data are deposited on Mendeley Data (https://data.mendeley.com/datasets/gvjd33sw57/2). The raw data associated with the Figs. 4, Fig. 6, Fig. 7, Fig. 8, Fig. 9, Fig. 10, Fig. 11, Fig. 12, Fig. 13, 15 and 16 can be consulted at Dataset 1 to Dataset 11, respectively.

## 2 Experimental Design, Materials and Methods

### 2.1 Materials

All chemicals used (Melamine, Formaldehyde (37%), Resorcinol, NaF, NaBr, NaOH, and HNO_3_) were analytical grade and supplied from CTR Scientific. Stock solutions and a calibration curve of fluoride and bromide were prepared by dissolving appropriate quantities of NaF and NaBr, respectively, in deionized water.

### 2.2 Synthesis of xerogels

Xerogels were synthesized using the methodology proposed by Muehlemann et al. [9]. Xerogels were firstly prepared by dissolving 4 g of melamine (M) in 20 mL of deionized water (W) and 15 mL of formaldehyde (F) in a glass flask under constant stirring for 5 min at 55 °C. Then, 10 mL of 0.5 M NaOH solution was added to the M—F—W mixture and maintained under agitation until a uniform transparent solution was obtained, next 3 mL of 37% HCl solution and 7 g of resorcinol were added, agitation was maintained for 1 more min; finally, the mixture was poured into a Petri dish glass which was placed inside an oven for 48 h at 55 °C. The molar ratio was fixed to M/R = 0.5. Additionally, two more xerogels were prepared with the same procedure, but increasing the molar ratio to M/R = 1.0 and M/R = 2.0, respectively, by adjusting the amounts of melamine, resorcinol, HCl but maintaining the 15 mL of formaldehyde.

Lastly, xerogel M/R = 0.5 was carbonized in a horizontal tubular furnace Carbolite model 12/65/550 under a N_2_ flow of 50 mL min^−1^ at 2 °C min-1 in the range of 55 to 115 °C, once the temperature of 115 °C was reached, it was kept for 30 min, then the temperature was increased to 400, 450 and 550 °C for 2 h on each temperature to finally obtain M/R = 0.5 400 °C, M/R = 0.5 450 °C, and M/R = 0.5 550 °C samples.

### 2.3 Adsorption experiments

The equilibrium adsorption experiments were carried out in a batch adsorber as describe elsewhere [10] with the following procedure: 0.02 g of carbonized xerogel sample was added to 50 mL of fluoride or bromide solution with different initial concentrations ranging from 10 to 100 mg L^−1^. The adsorber was partially immersed in a thermostatic water bath. The carbonized xerogel sample and the solution were kept in contact until the equilibrium was reached; previous tests demonstrated that the equilibrium was reached in 7 days. The experiments were carried out at constant pH; therefore, the pH was monitored and adjusted by adding few drops of 0.01, 0.1N NaOH, and HNO_3_ solutions. The mass of fluoride or bromide adsorbed per gram of adsorbent, (q), was obtained by using the following mathematical expression:

(1) $$q=\frac{V_{0}C_{0}-V_{f}C_{f}-\sum_{i=1}^{N}V_{i}C_{i}}{m}$$

(2) $$V_{f}=V_{0}-\sum_{i=1}^{N}V_{i}+V_{a}$$

Where: C_0_, C_f_, and C_i_ represent the initial concentration, final concentration, and the concentration of sample i of fluoride or bromide solution (mg/L); m is the mass of the adsorbent, (g); N is the number of samples; q is the mass of fluoride or bromide adsorbed per gram of adsorbent, (mg/g); V_0_, V_f_, V_i_, and V_a_ represent the initial volume, the final volume, the volume of sample i, and the total volume added of NaOH and HNO_3_ solutions to adjust pH, (L).

### 2.4 Measurement concentrations

The bromide and fluoride concentration in aqueous solution was measured by a potentiometric method with a bromide or fluoride ion-selective electrode. The method required a calibration curve of seven standard solutions (concentrations ranging from 0.6 to 14 mg/L).

## CRediT authorship contribution statement

**Nahum Andres Medellin-Castillo:** Conceptualization, Writing – original draft, Writing – review & editing. **Elizabeth Diane Isaacs-Páez:** Conceptualization, Writing – review & editing. **Liliana Giraldo-Gutierrez:** Writing – review & editing. **Juan Carlos Moreno-Piraján:** Writing – original draft. **Itzia Rodríguez-Méndez:** Visualization, Investigation. **Simón Yobanny Reyes-López:** Investigation. **Jaime Reyes-Hernández:** Investigation. **Sonia Judith Segovia-Sandoval:** Investigation.

## Declaration of Competing Interest

The authors declare that there is no conflict of interest.

## Data Availability

Data for the synthesis, characterization, and use of xerogels as adsorbents for the removal of fluoride and bromide in aqueous phase (Original data) (Mendeley Data).
